# GroEL1, from *Chlamydia pneumoniae*, Induces Vascular Adhesion Molecule 1 Expression by p37^AUF1^ in Endothelial Cells and Hypercholesterolemic Rabbit

**DOI:** 10.1371/journal.pone.0042808

**Published:** 2012-08-10

**Authors:** Chun-Yao Huang, Chun-Ming Shih, Nai-Wen Tsao, Yung-Hsiang Chen, Chi-Yuan Li, Yu-Jia Chang, Nen-Chung Chang, Keng-Liang Ou, Cheng-Yen Lin, Yi-Wen Lin, Chih-Hao Nien, Feng-Yen Lin

**Affiliations:** 1 Department of Internal Medicine, School of Medicine, College of Medicine, Taipei Medical University, Taipei, Taiwan; 2 Graduate Institute of Clinical Medicine, College of Medicine, Taipei Medical University, Taipei, Taiwan; 3 Research Center For Biomedical Implants and Microsurgery Devices, Taipei Medical University, Taipei, Taiwan; 4 Division of Cardiology, Department of Internal Medicine, Taipei Medical University Hospital, Taipei, Taiwan; 5 Division of Cardiovascular Surgery, Department of Surgery, Taipei Medical University Hospital, Taipei, Taiwan; 6 Graduate Institute of Integrated Medicine, China Medical University, Taichung, Taiwan; 7 Graduate Institute of Clinical Medical Sciences, China Medical University and Department of Anesthesiology, China Medical University Hospital, Taichung, Taiwan; 8 Cardiovascular Research Center, Taipei Medical University Hospital, Taipei, Taiwan; 9 Department of Computer Science and Information Management, Hung Kuang University, Taichung, Taiwan; Albert Einstein College of Medicine, United States of America

## Abstract

The expression of vascular adhesion molecule-1 (VCAM-1) by endothelial cells may play a major role in atherogenesis. The actual mechanisms *of chlamydia pneumoniae* (*C. pneumoniae*) relate to atherogenesis are unclear. We investigate the influence of VCAM-1 expression in the GroEL1 from *C. pneumoniae*-administered human coronary artery endothelial cells (HCAECs) and hypercholesterolemic rabbits. In this study, we constructed the recombinant GroEL1 from *C. pneumoniae*. The HCAECs/THP-1 adhesion assay, tube formation assay, western blotting, enzyme-linked immunosorbent assay, actinomycin D chase experiment, luciferase reporter assay, and immunohistochemical stainings were performed. The results show that GroEL1 increased both VCAM-1expression and THP-1 cell adhesives, and impaired tube-formation capacity in the HCAECs. GroEL1 significantly increased the VCAM-1 mRNA stability and cytosolic AU-binding factor 1 (AUF1) level. Overexpression of the p37^AUF1^ significantly increased VCAM-1 gene expression in GroEL1-induced bovine aortic endothelial cells (BAECs). GroEL1 prolonged the stability of VCAM-1 mRNA by increasing both p37^AUF1^ and the regulation of the 5′ untranslated region (UTR) of the VCAM-1 mRNA in BAECs. In hypercholesterolemic rabbits, GroEL1 administration enhanced fatty-streak and macrophage infiltration in atherosclerotic lesions, which may be mediated by elevated VCAM-1 expression. In conclusion, GroEL1 induces VCAM-1 expression by p37^AUF1^ in endothelial cells and enhances atherogenesis in hypercholesterolemic rabbits.

## Introduction

The first known case of infection with *Chlamydia pneumoniae* (*C. pneumoniae*) was a victim of sinusitis in Taiwan. *C. pneumoniae* is a Gram-negative bacterium that has a biphasic life cycle exhibiting both elementary and reticulate bodies. *C. pneumoniae* commonly causes pharyngitis, bronchitis and atypical pneumonia. There is extensive evidence indicating that *C. pneumoniae* may play a kind of key roles in the development of atherosclerosis [1]. Early fatty streaks show *C. pneumoniae* accumulation and that an increase in vascular intima-media thickness is associated with *C. pneumoniae* seropositivity [2]. *C. pneumoniae* may induce and accelerate the formation of atherosclerotic lesions in hypercholesterolemic C57BL/6J mice or ApoE3-Leiden mice [3]. During the infectious process of *C. pneumoniae*, elementary bodies (EBs) require both attachment to and phagocytosis by host cells [4]. Heat shock protein 60 from *C. pneumoniae* (also known as GroEL1) is also expressed on the surface of EBs and is the major binding protein of *C. pneumoniae.* GroEL1 can detach from the EBs and thus play an important role in the pathogenesis of infectious diseases [5]. In the clinical setting, GroEL1 causes respiratory tract and vascular diseases [6]. Additionally, GroEL1 may initiate the secretion of inflammatory cytokines, including interleukin (IL)-6, tumor necrosis factor (TNF)-α, IL-1β, and IL-8, as well as induce proliferation in vascular cells and mononuclear cells by triggering intracellular signaling pathway activation [7], [8], [9]. Recently, we have demonstrated that GroEL1 may increase the lectin-like oxidized low-density lipoprotein receptor-1 (LOX-1) expression and accelerate atherogenesis in rabbits on a high-cholesterol diet [10].

The critical role of adhesion molecules, intracellular adhesion molecule (ICAM)-1 and vascular adhesion molecule (VCAM)-1 in atherogenesis and plaque instability. Overexpression of VCAM-1 and ICAM-1 in plasma has been consistently observed in atherosclerotic lesion sites. Evidences support a crucial role for *C. pneumoniae* seropositivity, as it associated with a higher level of plasma ICAM-1 and VCAM-1 in the general population [11] and a higher level of plasma ICAM-1 and E-selectin in coronary arterial disease patients [12]. However, there are discrepancies among studies that report that *C. pneumoniae* causes the upregulation of ICAM-1 and VCAM-1 in human umbilical vein endothelial cells (HUVECs) [13] and the expression of ICAM-1 in human coronary artery endothelial cells (HCAECs) [14]. It is necessary to study the expression atherosclerosis-associated adhesion molecules that are affected by GroEL1 as a result of *C. pneumonuae* infection.

According to our recently finding that GroEL1 produced by *C. pneumoniae* increased neointimal hyperplasia in hypercholesterolemic rabbits, we hypothesized that GroEL1 would increase adhesion molecule expression (VCAM-1 and ICAM-1) in the endothelium. Therefore, we examined whether GroEL1 increased the expression of ICAM-1 and VCAM-1 in hypercholesterolemic rabbits. Furthermore, we explored the cellular events and the underlying mechanisms using HCAECs and bovine aortic endothelial cells (BAECs) *in vitro*.

## Materials and Methods

### Ethics Statement

All animals were treated according to protocols approved by the Institutional Animal Care Committee of the Taipei Medical University (Taipei, Taiwan). Experimental procedures and animal care conformed to the “Guide for the Care and Use of Laboratory Animals” published by the U.S. National Institutes of Health (NIH Publication No. 85–23, revised 1996).

### Construction of C. pneumonia GroEL1 Expression Vectors

*C. pneumoniae* (TWAR TW-183 from ATCC, VA, USA) was cultured in Hela 229 cells (from ATCC, VA, USA). The elementary bodies of *C. pneumoniae* were extracted according to a previous study [10]. The genomic DNA of *C. pneumoniae* was extracted from the elementary bodies using the EasyPure Genomic DNA mini kit (Bioman Scientific Co., Taipei, Taiwan). The segment containing the open-reading frame of GroEL1 was originally amplified by PCR with 100 ng of *C. pneumoniae* genomic DNA as a template, 0.2 mM dNTPs, 1 µM each of gene specific primers and 1 U Pfu DNA polymerase (Promega, Madison, WI, USA) using the following program: one cycle at 95°C for 5 min; 38 cycles at 95°C for 45 sec, 68°C for 45 sec, and 72°C for 2 min; 1 cycle at 68°C for 45 sec and 72°C for 10 min; and a final incubation at 72°C for 10 min with 1 U Taq DNA polymerase. The following gene specific primers were used in the PCR reaction: GroEL1 Pr-forward: 5′-CGAATTCTTAAGGAGAACAACGATGGCAG-3′ (forward primers contained an EcoRI site) and GroEL1 Pr-reverse: 5′-ACGGCCGGTAGTCCATTCCTGCGCTTGGC-3′ (reverse primers contained an EagI site). The amplified GroEL1 cDNA fragment was then cloned into a pCR2.1-TOPO vector (Invitrogen, Carlsbad, CA, USA) and was subsequently cloned in-frame into the EcoRI and NotI sites of a pGEX-5X-1 expression vector (GE Healthcare Amersham Biosciences, USA) for expression in *E. coli*. No new DNA sequence was generated in our study.

### Purification of the GroEL1 Recombinant Protein

BL21 cells (from Promega, WI, USA) were transformed with the pGEX-5X-1-GroEL1 expression vector, and recombinant GroEL1 was purified. Briefly, the BL21 cells containing the pGEX-5X-1-GroEL1 plasmid were grown overnight at 37°C in 2 mL LB medium supplemented with 100 µg/mL ampicillin. Then, 1.25 mL of the overnight culture was transferred into 100 mL of the LB/ampicillin medium and grown at 37°C to an A600 of 0.6–0.8 (approximately 2 h). Fusion protein expression was then induced by adding IPTG to a final concentration of 1 mM at 30°C for 6 h. Bacteria were pelleted by centrifugation for 10 min at 15000 g, and the recombinant GroEL1 was extracted under native conditions according to the GST Gene Fusion System manufacturer’s instructions (GE Healthcare Amersham Biosciences, USA). Finally, the recombinant GroEL1 protein was purified using an elution buffer containing 50 mM Tris-HCl and 10 mM reduced glutathione (pH 8.0). The quantity of recombinant GroEL1 was measured using the Bio-Rad Protein Assay (Bio-Rad, Hercules, CA, USA). The fusion protein was detected using SDS gel electrophoresis and was identified using immunoblotting with a GST antibody (GE Healthcare Amersham Biosciences, USA). Endotoxin levels in the recombinant GroEL1 were measured using a Limulus Amebocyte Lysate kit from Cambrex Inc. in the USA. LPS levels were below 1 pg/mL.

### Cell Culture

HCAECs and BAECs were purchased from Cascade Biologics, Inc. (Portland, OR, USA). Culture of the HCAECs and BAECs were performed according to the manufacturer’s instructions. Human monocytic THP-1 cells were purchased from ATCC (Manassas, VA, USA). THP-1 cells were grown in RPMI 1640 medium containing 5% FBS and were subcultured at a 1∶5 ratio three times per week.

### HCAECs/THP-1 Adhesion Assay

HCAECs (5×10^5^) were distributed into 24-well plates before the assay. Then, the growth medium was supplemented with the GroEL1 at the indicated concentrations for 24 h. The THP-1 cells were labeled for 1 h at 37°C with 10 µM of 2,7,-bis(2-carboxyethyl)-5(6)-carboxyflorescein acetoxymethyl ester (BCECF/AM, Boehringer-Mannheim) in serum-free RPMI 1640 media; they were then washed with PBS to remove free dye and resuspended in RPMI 1640 containing 2% FBS. One million labeled THP-1 cells were added to each HCAEC-containing well, and incubation continued for 1 h. Non-adherent cells were removed by three gentle washes with HBSS. The degree of MNC adhesion to the HCAECs was counted using Multilabel Counter Victor^2^ (Wallace, CA, USA) at an emission of 530 nm and an absorption of 435 nm after the cells were lysed with DMSO.

### Tube Formation Assay

A tube formation assay was performed on endothelial cells to assess the capacity for an endothelium barrier, which is believed to be important in vessel function. In brief, ECMatrix gel solution (Sigma-Aldrich, CA, USA) was thawed at 4°C overnight, mixed with ECMatrix diluent buffer and placed in a 96-well plate at 37°C for 1 h to allow the matrix solution to solidify. The HCAECs treated with or without GroEL1 for 48 h were harvested and replated (10,000 cells/well) on the top of the solidified ECMatrix gel in the culture medium supplemented with BSA (0.5%) and vascular endothelial growth factor (100 ng/mL). The cells were incubated at 37°C for 12 h. Tubule formation was inspected under an inverted light microscope (x100). Four representative fields were taken, and the average of the total area of complete tubes formed by the cells was compared using the computer software Image-Pro Plus.

### Western Blot Analysis

Total cell lysates or cytoplasmic fractions were extracted from the HCAECs. Proteins were separated using SDS–PAGE and were transferred to a PVDF membrane. The membranes were probed using mouse anti-VCAM-1 (R&D Systems, MN, USA), anti-ICAM-1 (R&D Systems, MN, USA), anti-human antigen R (HuR) (Santa Cruz Biotechnology, CA, USA), anti-AU binding factor 1 (AUF1) (Upstate Biotechnology, CA, USA) and anti-tristetraprolin (TTP) (Millopore, MA, USA) antibodies. The proteins were visualized using an enhanced chemiluminescence (ECL) detection kit (Amersham Biosciences, NJ, USA). Mouse anti-β-actin antibodies (Labvision/NeoMarkers, CA, USA) were used as loading controls.

### Cell Enzyme-linked Immunosorbent Assay (Cell ELISA)

To measure the cell-surface expression of adhesion molecules, the cell enzyme-linked immunosorbent assay (cell ELISA) was performed. The HCAECs in the 96-well plates were pretreated with GroEL1 at the indicated concentrations and durations. The expressions of cell-surface VCAM-1 and ICAM-1 were measured using separate incubations for 30 min at room temperature with goat antibodies against human VCAM-1 or ICAM-1 and then with horseradish peroxidase-conjugated rabbit anti-goat IgG for 1 h. The binding of the secondary antibody was verified by incubating the plates in the dark for 15 min with 3% *o*-phenylenediamine and 0.03% H_2_O_2_ in a 50 µM citrate buffer, and the reaction was terminated by adding 2 M H_2_SO_4_. The surface expression of adhesion molecules was quantified by reading the OD at 490 nm in an ELISA plate reader.

### Actinomycin D chase Experiment

Actinomycin D (20 µg/mL) was added to the cells for 1 h following the treatments under various experimental conditions. Total RNA was extracted at 0, 30, 60, 120, and 240 min after the addition of actinomycin D, and quantitative real time PCR was performed. The half-life (t_1/2_) of the TM mRNA was calculated according to the following formula t_1/2_ = 0.693/κ, where κ = ln (N_0_/N_t_)/t in which N_0_ is the cross-point of real-time PCR at t = 0 and N_t_ is the cross-point at time t.

### Immunohistochemical Staining

For immunofluorescent staining, cells were plated on cover slips, grown to confluence, and then treated with GroEL1 as indicated in the figure legends. After the treatment, the cells were fixed with 4% formaldehyde. Cell membranes were fenestrated using 0.4% Triton-100, and nonspecific binding sites were blocked with 2% BSA-Tween 20 (0.1% v/v). The cells were incubated with mouse anti-HuR, AUF1 or TTP (Chemicon, CA, USA) antibodies and then incubated with the secondary antibody conjugated to fluorescein isothiocyanate (FITC). The 2-(4-Amidinophenyl)-6-indolecarbamidine dihydrochloride (DAPI) was used to identify the nuclei. The slides were observed with fluorescent microscopy.

### Construction of AUF1 Isoform Expression Vectors

Plasmids for expression of each isoform of AUF1 were constructed as follows. Segments containing the open-reading frame of each isoform of AUF1s (p37^AUF1^, p40^AUF1^, p42^AUF1^, and p45^AUF1^) were originally amplified using THP-1 cells’ cDNA as a template, 0.2 mM dNTPs, 1 µM of each of gene-primers and 1 U Pfu DNA polymerase (Promega, Madison, WI, USA) with the following program: one cycle at 95°C for 5 min; 35 cycles at 95°C for 45 sec, 56°C for 45 sec, and 72°C for 70 sec; 1 cycle at 56°C for 45 sec, 72°C for 10 min; and a final incubation at 72°C for 10 min with 1 Unit Taq DNA polymerase. The following gene specific primers were used in the PCR: AUF1 Pr-forward: CTATGTCGGAGGAGCAGTTCG and AUF1 Pr-reverse: GCAATGGAATAATTTAGTATGGTTTGTAGC. The various amplified AUF1 cDNA fragments were cloned into pCR2.1-TOPO vectors (Invitrogen, Carlsbad, CA, USA) for sequencing and were subsequently cloned in-frame into the EcoRI protein site of the pcDNATM 4/His A (Invitrogen, Carlsbad, CA, USA) vector for expression in mammalian cells. No new DNA sequence was generated in our study.

### Construction of Luciferase Reporter Plasmids Containing 5′ or 3′ UTR of VCAM-1 mRNA

To generate a CMV promoter-derived luciferase reporter plasmid for expression in mammalian cells, segments containing the open-reading frame of the luciferase were digested from pGL3-Basic Vector (Promega, Madison, WI, USA) using NheI and XbaI restriction enzymes and then cloned into the same sites of pcDNA™3.1/myc-His(-) A (Invitrogen, Carlsbad, CA, USA). For the construction of the luciferase reporter plasmids containing 5′ or 3′ untranslated regions (UTR) of VCAM-1 mRNA, segments containing the 5′ or 3′ UTR of VCAM-1 mRNA were originally amplified using PCR with 100 ng of human genomic DNA as a template. The following specific primers were used in the PCR reaction: VCAM-1-5′UTR Pr-forward: CGCTAGCAAACTTTTTTCCCTGGCTCTGC and VCAM-1-5′UTR Pr-reverse: GGCTAGCTTAAGTCCTCCGTGGTGATGAG (both primers contained a NheI site); VCAM-1-3′UTR Pr-forward: ctaatgcttgatatgttcaactgg and VCAM-1-3′UTR Pr-reverse: ctaattccagaaattatcactttactatac. The amplified 5′ or 3′ UTR of VCAM-1 mRNA fragments were then cloned into the pCR2.1-TOPO vector (Invitrogen, Carlsbad, CA, USA) for sequencing and then cloned into the NheI (5′ of luciferase reporter) or EcoRI site (3′ of luciferase reporter) of the CMV promoter-derived luciferase reporter plasmid, respectively. No new DNA sequence was generated in our study.

### Luciferase Reporter Assay

Functional analysis of the 3′ and 5′ UTR of VCAM-1 mRNA was performed using plasmids containing the 3′ or 5′ UTR and the luciferase reporter gene. One million BAECs were trypsinized and resuspended in 100 µl of Nucleofector solution; 1 µg of the reporter plasmid was transfected using the Nucleofector electroporation device according to the manufacturer’s instructions. Equal amounts of the pcDNA™3.1 vector (mock plasmid) or the luciferase reporter gene contained pcDNA™3.1 plasmid (CMV-Luciferase) were used as control groups. Uniform transfection efficiencies were normalized using a β-galactosidase reporter plasmid. Cell extracts were prepared with reporter lysis buffer (300 µl/well), protein concentrations were determined, and the luciferase activity was quantified using luminometry (Wallac Victor^2^, Finland) using the luciferase assay system (Promega, Madison, WI, USA). β-galatosidase activity was measured using a β-galactosidase enzyme assay kit (Stratagene, La Jolla, CA, USA).

### Animal Experiment

The animal treatment protocol was established according to a previous study [10]. 25 adult male New Zealand white rabbits (2.5–3 kg) were used. After 1 week on a commercial rabbit chow diet (Scientific Diet Services, Essex, UK) at 60 g/kg per day with water *ad libitum*, 15 animals were placed on a 2% high-cholesterol (HC) diet (Purina Mills Inc., St. Louis, MO, USA) and 10 animals were placed on a normal diet. The animals were divided into 5 groups (5 animals in each group): group 1 was the control; group 2 received the HC diet; group 3 received the HC diet and intravenous injections of GroEL1 (2 µg/kg of body weight) through the ear vein at the end of weeks 2, 3, and 4; group 4 received the HC diet and intravenous injections of GroEL1 (4 µg/kg of body weight) at the end of weeks 2, 3, and 4; and group 5 received the normal chow diet and GroEL1(4 µg/kg of body weight) at the end of weeks 2, 3, and 4. The animals were sacrificed at the end of week 4, and the abdominal aortas were harvested, gently dissected free of adherent tissues, rinsed with ice-cold phosphate buffered saline, immersion-fixed with 4% buffered paraformaldehyde, paraffin-embedded, and cross-sectioned in 5-µm-thick serial sections for immunohistochemistry using anti-VCAM-1, anti-ICAM-1, anti-RAM-11, anti-AUF1, anti-HuR, and anti-TTP antibodies. The slides were observed using light microscopy.

Images were subjected to statistical evaluation of immunohistochemical positively stained cells by unbiased estimation in 10 random fields of view at a magnification of × 400. Average numbers of positively stained cells per high power field (HPF) were provided.

### Statistical Analysis

Results were expressed as the mean ± SEM. Data were analyzed using ANOVA followed by the Dunnett’s test. A *p* value less of than 0.05 was considered statistically significant.

## Results

### VCAM-1 Mediates the Binding of the THP-1 Cells to GroEL1-stimulated HCAECs

**Figure 1:**
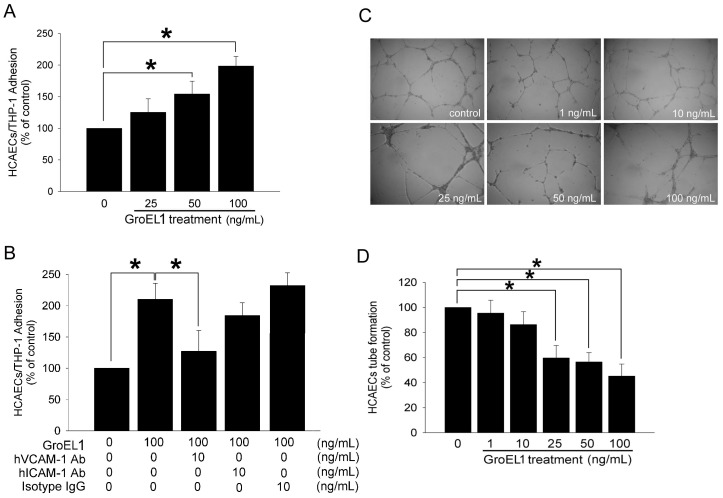
GroEL1 induces the binding of HCAECs/THP-1 cells and impairs the tube formation capacity of the HCAECs. (A) HCAECs were pretreated with 25–100 ng/mL of GroEL1 for 24 h and then co-cultured with THP-1 cells for 1 h. (B) Cells were pretreated with anti-hVCAM-1 or anti-hICAM-1 antibodies for 30 min, followed by GroEL1 treatment for 24 h. HCAECs and THP-1 cells were co-cultured for 1 h. The degree of THP-1 adhesion to the HCAECs was counted using a Multilabel Counter Victor^2^. The isotype IgG was as a negative control. (C) HCAECs were pretreated with 1–100 ng/mL of GroEL1 for 48 h. An *in vitro* tube formation assay was performed using ECMatrix gel to investigate the effect of GroEL1 on the HCAECs’ lining function. Representative photos for *in vitro* tube formation are shown. (D) The bar graph demonstrates the tube formation capacity of the GroEL1-treated HCAECs. All data represent the results of three independent experiments (mean ±SD; *P<0.05 was considered significant and n = 3).

In figure 1A, the confluent control HCAECs showed minimal binding of the THP-1 cells (1538±680 cells), but adhesion was substantially increased when the HCAECs were treated with 50 or 100 ng/mL of GroEL1 for 24 h. Adhesion molecules mediate the adhesion of monocytes to the endothelium, we predict that VCAM-1 and ICAM-1 may be involved in this process. The treatment with goat anti-hVCAM-1 antibody on the HCAECs after 100 ng/mL of GroEL1 treatment significantly reduced the adhesion of the THP-1 cells (Figure 1B). In contrast, pretreatment with goat anti-hICAM-1 antibody or isotype IgG on HCAECs did not reduce the adhesion of the THP-1 cells to the GroEL1-stimulated HCAECs. These results indicate that GroEL1 induces THP-1 cell/HCAECs adhesion mediated by the HCAECs’ VCAM-1 expression.

### GroEL1 Treatment Impairs the Tube Formation Capacity of HCAECs

In figure 1C and D, the HCAECs successfully generate a capillary network on Matrigel. After 48 h of culturing in 25–100 ng/mL of GroEL1, the functional capacity for tube formation of the HCAECs on ECMatrix gel was significantly decreased compared to the control group. The results indicate that concentrations of more than 25 ng/mL of GroEL1 decrease the capacity of tube formation, which are involved in the lining function of HCAECs.

### GroEL1 Induces the VCAM-1 Expression and Increases VCAM-1 mRNA Stability on HCAECs

**Figure 2:**
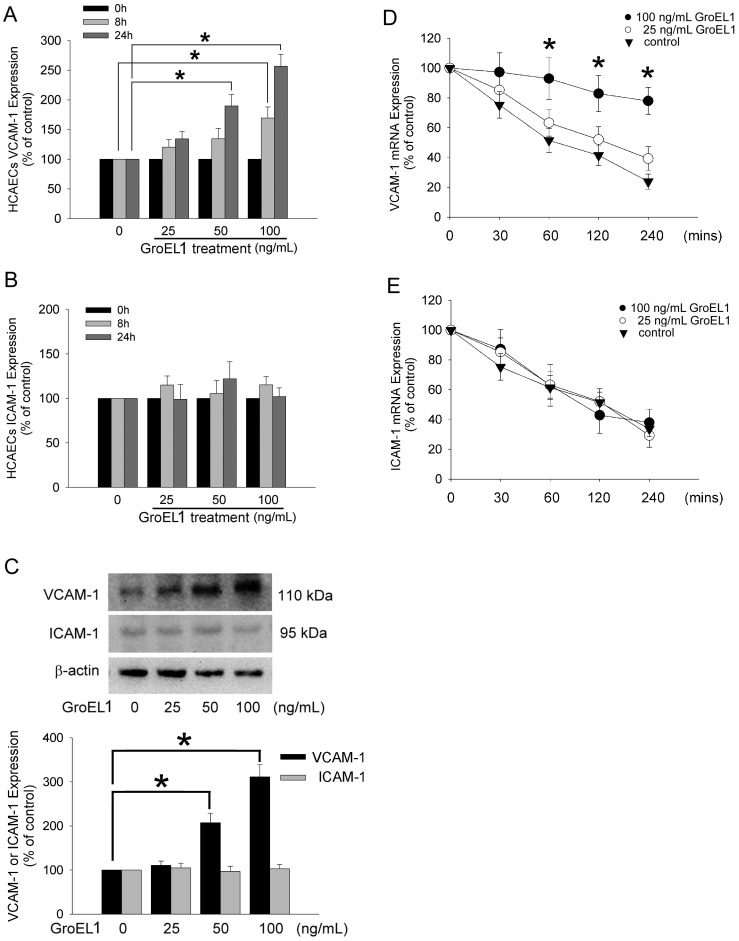
GroEL1 induces VCAM-1 expression and increases VCAM-1 mRNA stability on the HCAECs. Cells were treated for 8 or 24 h with 25–100 ng/mL of GroEL1. (A and B) Expression of VCAM-1 and ICAM-1 were detected by cell ELISA. All data represent the results of three independent experiments (mean ±SD; *P<0.05 was considered significant and n = 3). (C) Cells were stimulated for 24 h with 25–100 ng/mL of GroEL1. Western blot analyses of VCAM-1 and ICAM-1 proteins were performed. The total β-actin protein was used as loading control. The bar graph shows the quantified results using densitometry. (D and E) Actinomycin D chase experiment was performed to evaluate the stability of the VCAM-1 mRNA and ICAM-1 mRNA. Cells were treated with 25 ng/mL (○) or 100 ng/mL (•) of GroEL1 before actinomycin D treatment for 40 minutes. (▾) The stability of VCAM-1 mRNA was demonstrated in naïve cells. Total RNA was extracted at various time points and quantitative real-time PCR was performed. All data represent the results of three independent experiments (mean ±SD; *P<0.05 was considered significant compared to control and n = 3).

GroEL1-induced adhesion molecule expression caused a significant dose- and time-dependent increase in VCAM-1 expression compared to naïve HCAECs (Figure 2A). In contrast, GroEL1 had no significant effect on ICAM-1 expression (Figure 2B). To confirm these findings, western blot analysis was performed (Figure 2C). The amounts of VCAM-1 and ICAM-1 were low in the control (untreated) HCAECs. GroEL1 treatment (50 or 100 ng/mL) for 24 h caused significant up-regulation in expression of VCAM-1 but not ICAM-1. Controlling the stability of the VCAM-1 and ICAM-1 mRNA modulates gene expression and efficiently adjusts inflammatory responses. To determine whether GroEL1 affects the steady-state dynamic balance between the rate of transcription and the message stability of the VCAM-1 and ICAM-1 mRNA, an actinomycin D chase experiment was conducted. The mRNA half-life, deduced under various conditions according to the curve, indicated that 100 ng/mL of GroEL1 stimulation rapidly increased the stability of the VCAM-1 mRNA in HCAECs (half life of VCAM-1 mRNA: 25 ng/mL GroEL1 group, 125.2±31.4 min; 100 ng/mL GroEL1 group, more than 240 min; control group, 90.6±25.1 minutes; Figure 2D). The addition of GroEL1 did not change the stability of the ICAM-1 mRNA in HCAECs. These results indicate that GroEL1 induces VCAM-1 protein expression and prolongs VCAM-1 mRNA stability in HCAECs.

### GroEL1 Treatment Induces p37^AUF1^, p40^AUF1^, and p42^AUF1^ Activation

**Figure 3:**
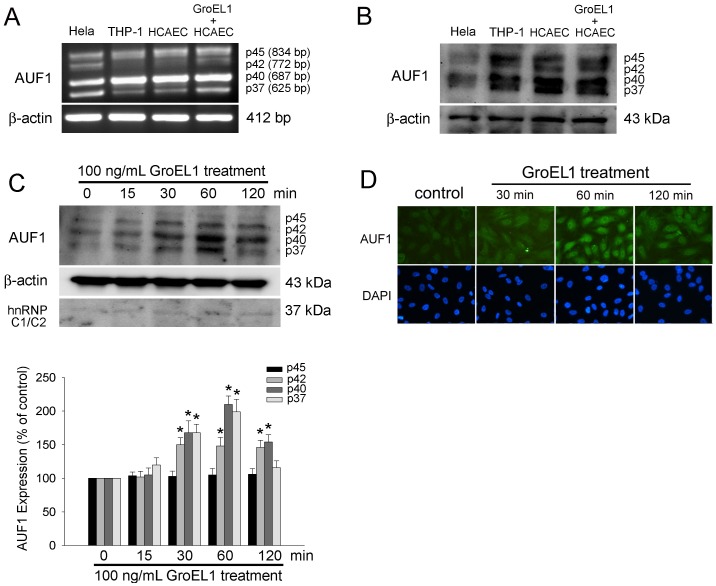
GroEL1 treatment induces AUF1 activation. (A) HCAECs were treated with 100 ng/mL of GroEL1 for 60 minutes. The total mRNA was extracted from cells. Traditional RT-PCR was performed for the four subunits of AUF1 expression. The β-actin mRNA was used as a internal control. (B) HCAECs were treated with 100 ng/mL of GroEL1 for 60 minutes. The total protein was extracted from cells. Western blot analysis was performed for the four subunits of AUF1 expression. The β-actin protein was used as a loading control. The endogenous total mRNA and protein extracted from the Hela or THP-1 cells was used to confirm the efficiency of the primer and antibodies. (C) HCAECs were treated with 100 ng/mL of GroEL1 for 15–120 minutes. The cytosolic level of AUF1 was analyzed using western blotting. The β-actin and hnRNP C1/C2 protein was used as a loading control. The density of band was quantified using densitometry and showed as bar graph. Data are expressed as % of control, presented as the mean ± SEM and represent the results of three independent experiments (n = 3, *P<0.05 was considered significant compared to control at the same group). (D) The intracellular AUF1 was identified using immunocytofluorescence and observed with a fluorescent microscope. DAPI was used to stain the nuclei of the HCAECs.

Previous evidence demonstrated that the mRNA-binding protein AUF1 mediates the expression of inflammatory-related genes [15]. The traditional RT-PCR and western blotting were used to measure the AUF1 mRNA and protein expression in the HCAECs. The Hela and THP-1 cells express the four isoforms of AUF1 endogenously; therefore, the total mRNA and protein were used to confirm the efficiency of the primer and antibody. Figure 3A and B show that the AUF1 mRNA and protein was found predominantly in naïve Hela and THP-1 cells. The accumulation of p37^AUF1^, p40^AUF1^, p42^AUF1^, and p45^AUF1^ was observed both in the 100 ng/mL of GroEL1-treated and naïve HCAECs. When treated with 100 ng/mL of GroEL1 for 15–120 minutes, the levels of p37^AUF1^, p40^AUF1^, and p42^AUF1^ increased over time. The intracellular level of p42^AUF1^, p40^AUF1^, and p37^AUF1^ reached a maximum after stimulation at 60 minutes. In contrast, the level of p45^AUF1^ remained unchanged following the GroEL1 treatment (Figure 3C). The heterogeneous nuclear ribonucleoprotein (hnRNP) C1/C2 and β-actin were used to supervise the process of protein extraction. Additionally, a fluorescent microscope was used to observe the distinct effect of GroEL1 on the HCAECs. Figure 3D shows that GroEL1 triggers a distinct increase in cytoplasmic AUF1 in HCAECs after 60 minutes. These observations suggest that GroEL1 treatment significantly increases cytoplasmic p42^AUF1^, p40^AUF1^ and p37^AUF1^ subunit accumulation but does not influence the level of p45^AUF1^.

### p37^AUF1^ Subunit Activation Mediates VCAM-1 mRNA Stability and 5′UTR of VCAM-1 mRNA Confer GroEL1 Responsiveness

**Figure 4:**
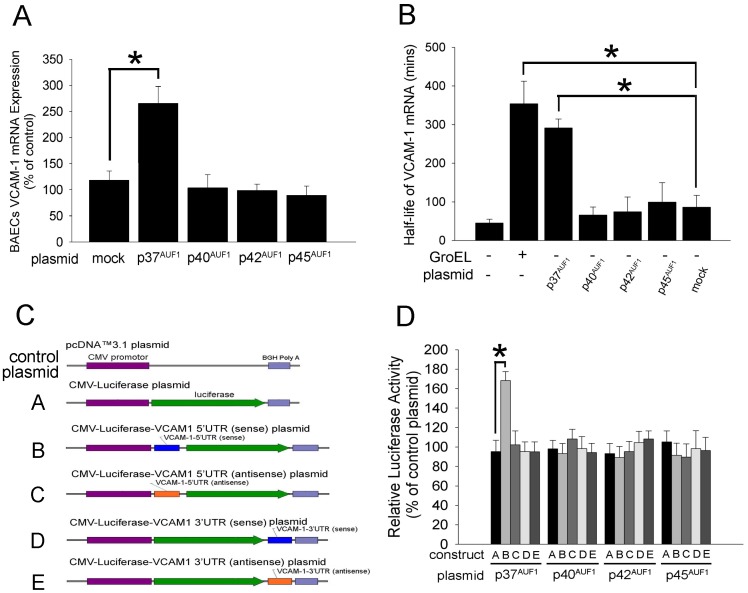
The 5′ UTR flanking sequence of VCAM-1 mRNA conferred P37^AUF1^-responsiveness in the BAECs. (A) The BAECs were transfected with the 4His-A-AUF1-p37 plasmid (p37^AUF1^), 4His-A-AUF1-p40 plasmid (p40^AUF1^), 4His-A-AUF1-p42 plasmid (p42^AUF1^), or 4His-A-AUF1-p45 plasmid (p45^AUF1^). The level of VCAM-1 mRNA were analyzed using real-time PCR after transfectiuon for 24 hours. (B) The VCAM-1 mRNA stability was analyzed using an actinomycin D chase experiment in the AUF1-transfected BAECs. (C) Schematic representation of the various plasmids containing the luciferase and UTR of the VCAM-1 mRNA. Control plasmid: pcDNA™ 3.1 plasmid; construct A, CMV-Luciferase plasmid; construct B, CMV-Luciferase-VCAM1 5′UTR (sense) plasmid; construct C, CMV-Luciferase-VCAM1 5′UTR (antisense) plasmid; construct D, CMV-Luciferase-VCAM1 3′UTR (sense) plasmid; construct E, CMV-Luciferase-VCAM1 3′UTR (antisense) plasmid. (D), BAECs were co-transfected with the CMV-Luciferase-VCAM1 UTR plasmid, the β-galactosidase reporter plasmid, and the 4His-A-AUF1 plasmid. Uniform transfection efficiencies were confirmed using a β-galactosidase reporter plasmid. The luciferase activity was quantified by luminometry. Data are expressed as relative luciferase units, presented as the mean ± SEM and represent the results of three independent experiments (*P<0.05 was considered significant and n = 3).

To explore which subunit of AUF1 regulates VCAM-1 mRNA expression and stability, we constructed AUF1 expression vectors, including 4His-A-AUF1-p37 plasmid, 4His-A-AUF1-p40 plasmid, 4His-A-AUF1-p42 plasmid, and 4His-A-AUF1-p45 plasmid. Figure S6 shows the expression of the plasmids in BAECs. Real time PCR demonstrated higher levels of VCAM-1 mRNA in the 4His-A-AUF1-p37 plasmid-transfected group than in the His-A-AUF1-p40 plasmid-, 4His-A-AUF1-p42 plasmid-, or 4His-A-AUF1-p45 plasmid-transfected groups (Figure 4A). Furthermore, the actinomycin D chase experiment showed that the transfection of 4His-A-AUF1-p37 plasmid rapidly increased the stability of the VCAM-1 mRNA in the BAECs (half life of VCAM-1 mRNA: in naïve BAECs group, 45.3±9.5 minutes; in 4His-A-AUF1-p37 plasmid group, 291.3±23.0 minutes; Figure 4B). The addition of situation did not change the stability of the VCAM-1 mRNA in the BAECs. To investigate whether the UTR promotes VCAM-1 mRNA expression, a reporter plasmid containing the 3′ or 5′_UTR and the luciferase reporter gene were transfected into the BAECs. A schematic representation of the various plasmids containing the luciferase and the UTR of the VCAM-1 mRNA are shown in Figure 4C. The CMV-Luciferase-VCAM1 5′ UTR (sense) plasmid plus 4His-A-AUF1-p37 plasmid cotransfected group had a higher luciferase activity than the control groups (pcDNA™3.1 plasmid-transfected cells). In contrast, other cotransfected plasmids (CMV-Luciferase-VCAM1 5′ UTR (antisense) plasmid, CMV-Luciferase-VCAM1 3′ UTR (sense) plasmid, and CMV-Luciferase-VCAM1 3′ UTR (antisense) plasmid) as well as the 4His-A-AUF1 plasmid did not increase the activity of luciferase in the BAECs (Figure 4D). These findings suggest that the 5′ UTR of VCAM-1 mRNA confers the GroEL1 responsiveness and that p37^AUF1^ modulates the 5′ UTR-mediated gene expression in endothelial cells.

### GroEL1 Enhances Infiltration of Macrophages and Induces VCAM-1 and AUF1 Expression in Rabbit Aorta

Similar to previous demonstrated [10], control rabbits and the HC diet groups did not have a thickened intima or atherosclerotic lesion formation in the abdominal aortas. Abdominal aortas had slight lesion formation in the HC diet +2 µg/kg BW GroEL1 group and marked lesion formation in the HC diet +4 µg/kg BW GroEL1 group compared with these areas in the control group. Staining with the anti-RAM-11 antibody to identify infiltrated macrophages showed that fewer macrophages infiltrated into the vessel walls in the control and HC diet groups compared with the HC diet + GroEL1 groups. Although the 4 µg/kg BW GroEL1 group did exhibit an increased macrophage infiltration (data not shown), there was a visible GroEL1 level-dependent severity of the atherosclerotic lesions in the HC diet rabbits.

**Figure 5:**
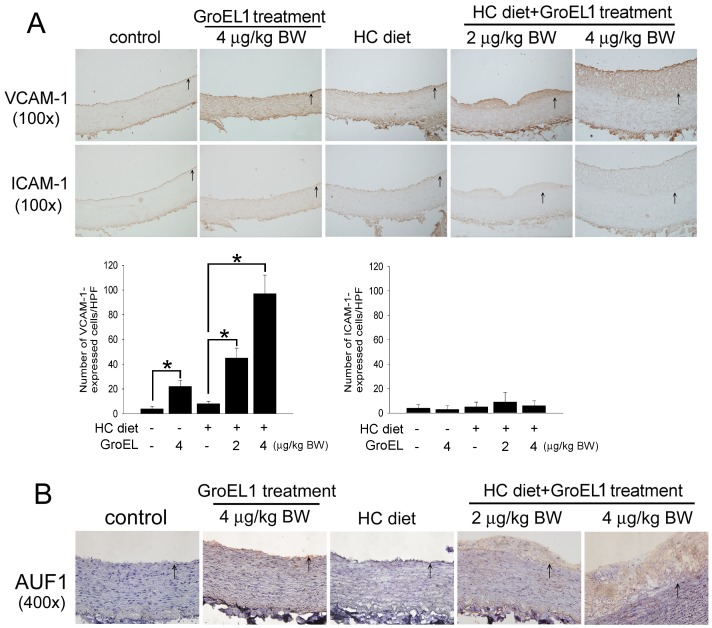
GroEL1 induces VCAM-1 and AUF1 expression in rabbits. (A) Immunohistochemistry to assess the VCAM-1 and ICAM-1 expression in the rabbit abdominal aorta. Corresponding hematoxylin staining was used for nucleus identification. The intima was markedly thickened in the GroEL1+HC diet treatment groups compared with the control, GroEL1 treatment, and HC diet treatment groups. The graphs show 100× magnification of the slide. Quantifications of immunohistochemical positively stained cells were shown in the lower panel. (B) Immunohistochemistry to assess AUF1 expression in the rabbit abdominal aorta. Compared with the control and HC diet treatment groups, AUF1 staining was observed on the luminal surface in the 4 µg/kg BW GroEL treatment group. Positive AUF1 staining in the thickened intima was observed in the 2 µg/kg BW GroEL1+HC diet and 4 µg/kg BW GroEL1+HC diet treatment groups. The graphs show a 100× magnification of the slides. The lumen is uppermost in all sections, and the internal elastic laminae is indicated by the arrows.

According to the results *in vitro* that GroEL1 induces VCAM-1 expression and AUF1 activation in HCAECs and *in vivo* that GroEL1 enhances macrophage infiltration in HC diet-fed rabbits, we analyzed the adhesion molecule (VCAM-1 and ICAM-1) expression and the RNA binding proteins (AUF1, HuR, and TTP) in GroEL1 treated rabbits. Figure 5A shows that increasing VCAM-1 expression is observed on the vessel wall in only the GroEL1-treated groups compared to the control and HC diet-fed groups. Indeed, the combination of the GroEL1 treatment and the HC diet may prove that the VCAM-1 expression is increased only in the HC diet-fed group. In contrast, the ICAM-1 expression was not affected by the GroEL1 treatment or the HC diet in rabbits. To confirm the finding that the GroEL1 administration affects AUF1 expression, which is associated with VCAM-1 expression, immunohistochemical staining was performed on sections of the abdominal aortas (Figure 5B). Compared with sections from the control group, the sections of the abdominal aorta showed a slightly thickened intima in the 4 µg/kg BW GroEL treatment group, a markedly thickened intima in the HC diet +2 µg/kg BW GroEL treatment group, and severe intimal hyperplesia in the HC diet +2 µg/kg BW GroEL treatment group. Compared with the control and HC diet treatment groups, AUF1 staining was observed on the luminal surface in the 4 µg/kg BW GroEL treatment group. Strong, positive AUF1 staining in the markedly thickened intima was observed in the 2 µg/kg BW GroEL1+HC diet and 4 µg/kg BW GroEL1+HC diet treatment groups. The expression of HUR and TTP remained unchanged following the GroEL1 treatment and HC diet in rabbits’ aorta (Figure S1). These results demonstrate that GroEL1 administration increased AUF1 expression (GroEL1 treatment group versus control group) and significantly decreased AUF1 expression (GroEL1+HC diet treatment group versus the GroEL1 treatment group only) in the vessel wall.

## Discussion

GroEL1 from *C. pneumoniae* impaired the lining function and induced VCAM-1 expression, which mediates monocytes adhesion and infiltration. The p37^AUF1^ isotype of AUF1 interacts with the 5′ UTR of the VCAM-1 mRNA to prolong the stability of the VCAM-1 mRNA in GroEL1-stimulated endothelial cells. Additionally, the GroEL1 increased VCAM-1 expression, monocyte infiltration, and neointimal hyperplasia in hypercholesterolemic rabbits. In fact, the administration of the GroEL1 also increased the AUF1 expression in the vessel wall of hypercholesterolemic rabbits. The data provide evidence for a direct involvement of GroEL1, which may enhance VCAM-1 expression in the endothelium and may mediate atherosclerosis progression.

Clinical results have demonstrated that there is detectable *C. pneumoniae* in hypertropic human coronaries using immunohistochemistry, which may affect the lesion progression [16]. The seropositivity of *C. pneumoniae* was associated with a higher plasma level of ICAM-1 and VCAM-1 [11]. In 2008, Nicole Borel *et al*. first demonstrated evidence of persistent GroEL1 from *C. pneumoniae* in human coronary atheromatous [6], even though the meta-analyses of randomized clinical trials of antibiotic therapy for secondary prevention of coronary heart disease did not show any benefits [17], [18]. Additionally, we have also demonstrated the potential role of *C. pneumoniae* GroEL1 in atherosclerosis induction in the presence of hypercholesterolemia [10]. The GroEL1 that resembles the reticulate bodies of *C. pneumoniae* [19], [20] may mediate the cellular inflammatory responses [6], [21]; this response may be a potential rationale for the dramatic failure of clinical trials using antibiotics for secondary prevention of coronary artery diseases. It has recently been shown that *C. pneumoniae* heat shock protein 60 can activate human vascular endothelia cells, smooth muscle cells, and macrophages [7], stimulate cellular LDL oxidation *in vitro* [22], and regulate macrophage TNF-α and matrix metalloproteinase expression [23]. In fact, *C. pneumoniae* infection induced cytokines, E-selectin, ICAM-1, and VCAM-1 expression in endothelial cells [13], [14]; the patients suffering from coronary artery disease had a higher plasma level of ICAM-1 and E-selectin, which was associated with *Chlamydia* IgA LPS seropositivity [12]. In our study, we first show that the administration of GroEL1 from *C. pneumoniae* significantly increased VCAM-1 expression in HCAECs, which may be mediated by post-transcriptional modification. In contrast, GroEL1 does not affect the expression of ICAM-1 in HCAECs. We also speculate that the other components, except GroEL1 from *C. pneumoniae,* may contribute to the ICAM-1 expression in endothelial cells resulting in atherosclerosis.

The activation of endothelial cells and smooth muscle cells play key roles during atherogenesis. In particular, nuclear factor κB (NF-κB) is essential which is triggering the inflammatory responses of vascular cells. However, previous studies had demonstrated that GroEL1 of *C. pneumoniae* may induce phosphorylation of ERK1/2 mitogen-activated protein kinase [8] and activation of NF-κB [7], [21] in cells following infection of *C. pneumonia*. In addition to NF-κB-related transcriptional pathways, the mechanisms of post-transcriptional modification, which regulate the stability of the mRNA transcripts, are also associated with inflammation [15], [24]. The stability of the mRNA is often modulated by AU-rich elements (AREs) through untranslated regions (UTR) [25]. A number of RNA binding proteins bind with AREs, including AUF1, HuR, and TTP. HuR binds to AREs and stabilizes thrombomodulin [26], granulocyte macrophage colony stimulating factor (GM-CSF), c-Fos, and TNF-α [27], [28], [29]. In contrast, TTP destabilized TLR4, [30] thrombomodulin [26], TNF-α [31], GM-CSF [32], and IL-3 [33] mRNA. However, the activation of AUF1 causes diverse results in mRMA stability during inflammation [34], [35], [36], [37]. In this study, TTP and HUR were found predominantly in nontreated HCAECs. The western blot analysis and fluorescent microscopy demonstrated that the expression level and distribution of HUR and TTP remained unchanged following the GroEL1 treatment (Figure S2). The p37^AUF1^, p40^AUF1^, p42^AUF1^, and p45^AUF1^ isoforms are derived from the AUF1 gene by alternate splicing. Each isoform does not contain a unique sequence; therefore, a siRNA approach cannot be used to knock down specific AUF1 isoform expression. Therefore, we generate 4 AUF1 isoform expression plasmids, to identify the roles involved in post-transcriptional modification of VCAM-1 mRNA. We have shown the sequence of the 3′ and 5′_UTR of VCAM-1 mRNA in Figure S3. However, the 3′ UTR, but not the 5′ UTR of the VCAM-1 mRNA, mainly contains AU motifs. Although our data provide evidence that p37^AUF1^ is an essential and critical regulator of VCAM-1 expression via regulation of the 5′UTR of mRNA, we still cannot provide evidence of the minimal region and motif requirement for the nucleotides in this interaction. In the future, we plan to study serially deleted constructs and electrophoretic mobility shift assay (EMSA) to identify the minimal region required for the 5′ UTR of the VCAM-1 mRNA interaction with p37^AUF1^.

VCAM-1 is a protein located on the endothelial cell surface involved with the binding of monocytes, which participate in atherogenesis. In 2001, Myron I. Cybulsky demonstrated that VCAM-1, but not ICAM-1, plays a dominant role in the initiation of atherosclerosis [38]. However, the increasing in VCAM-1 protein expression in cells is a consequence of accumulated VCAM-1 mRNA. Except for the regulation of transcriptional signaling pathways, this might occur because of the enhanced VCAM-1 mRNA stability mediated by regulation of post-transcriptional modifications [39], [40]. IL-4, IL-13, and TNF-α induced the elevation of VCAM-1 expression in human cells resulting from the stabilization of the VCAM-1 mRNA [39], [41]. Nevertheless, there is little evidence to explain how the post-transcriptional mechanisms, involving RNA processing or mRNA stability, may play in VCAM- 1 gene expression, as has been demonstrated for ICAM-1 [42], [43]. C. Frank Bennett *et al.* showed that oligonucleotides hybridized to the 3′ UTR of VCAM-1 mRNA and promoted a reduction in the respective mRNA levels [44]; Tamia A Harris *et al*. defined that microRNA-126 binds to the 3′ UTR of VCAM-1 mRNA and suppresses the stability and expression of VCAM-1 mRNA [45] in endothelial cells. Until now, there have been many doubts about the mechanisms of regulation of the 5′ UTR of VCAM-1 mRNA. In fact, we are the first group to explore the VCAM-1 expression via the regulation of the 5′ UTR of mRNA and p37^AUF1^ activation in GroEL1-stimulated HCAECs in spite of the advanced experiments needed to perform.

In this study, the BAECs used for the luciferase reporter assay resulted from a low efficiency of plasmid cotransfection in the HCAECs. Indeed, in the early stages of the study, we had also performed the test using BAECs to analysis whether BAECs have responses to GroEL1 treatment similarly to HCAECs. The results showed that GroEL1 induces the binding of BAECs/THP-1 cells and impairs the tube formation capacity of the BAECs (Figure S4). In the BAECs, only the p40^AUF1^ isotype existed (NP_001091533). However, there is a high sequence homology (approximately 99%) between the HCAECs’ p40^AUF1^ protein and BAECs’ p40^AUF1^ protein. Additionally, we have shown the results of increasing of the VCAM-1 mRNA expression and stability in the GroEL-stimulated BAECs in Figure S5. When the sequence of the BAECs VCAM-1 3′UTR or 5′UTR is replaced with that in HCAECs, approximately 78% homology was observed. The CMV-luciferase-VCAM-1 5′ UTR (sense) plasmid plus the 4His-A-AUF1-p37 plasmid cotransfected group had a higher luciferase activity than the control groups in the BAECs, indicating that the post transcriptional regulation is in the cells. Although it was not fully appropriate for the expression of human AUF1 and VCAM-1 mRNA UTR plasmids in the BAECs as compared with the HCAECs, we believe that the results of this experiment are still important and credible. In conclusion, our results demonstrate that *C. pneumoniae* GroEL1 may induce VCAM-1 expression in endothelial cells, which mediates fatty streak formation in atherogenesis. The elevated level of VCAM-1 may be mediated by post-transcriptional modification. Our work identified VCAM-1 as a target gene for GroEL1. This provides a basis for further investigation of VCAM-1 modulation as a therapeutic strategy for atherogenesis in *C. pneumoniae* infection.

## Supporting Information

Figure S1 **Immunohistochemistry to assess the HuR and TTP expression in the rabbit abdominal aorta.**
(DOC)

Figure S2 **Fluorescent microscopy and western blot analysis demonstrated that the expression level and distribution of HUR and TTP remained unchanged following GroEL1 treatment.**
(DOC)

Figure S3 **The sequence of the 3′ and 5′_UTR of the VCAM-1 mRNA.**
(DOC)

Figure S4 **GroEL1 induces the binding of BAECs/THP-1 cells and impairs the tube formation capacity of the BAECs.**
(DOC)

Figure S5 **GroEL1 increases the production of VCAM-1 mRNA and prolongs the stability of the VCAM-1 mRNA in the BAECs.**
(DOC)

Figure S6 **The expression of the 4His-A-AUF1-p37 plasmid, 4His-A-AUF1-p40 plasmid, 4His-A-AUF1-p42 plasmid, and 4His-A-AUF1-p45 plasmid in BAECs.**
(DOC)
