# Correction: GroEL1, from *Chlamydia pneumoniae*, Induces Vascular Adhesion Molecule 1 Expression by p37^AUF1^ in Endothelial Cells and Hypercholesterolemic Rabbit

**DOI:** 10.1371/annotation/6f8adaaa-19d8-4187-9418-24a05ae77c8c

**Published:** 2012-08-20

**Authors:** Chun-Yao Huang, Chun-Ming Shih, Nai-Wen Tsao, Yung-Hsiang Chen, Chi-Yuan Li, Yu-Jia Chang, Nen-Chung Chang, Keng-Liang Ou, Cheng-Yen Lin, Yi-Wen Lin, Chih-Hao Nien, Feng-Yen Lin

The following information was missing from the Funding section: This work was also funded by Taipei Medical University (TMU100-AE3-Y15).

