# Supplementary material for: GroEL1, from Chlamydia pneumoniae, Induces Vascular Adhesion Molecule 1 Expression by p37AUF1 in Endothelial Cells and Hypercholesterolemic Rabbit
Source: PLoS One. 2012 Aug 10;7(8):e42808. doi: 10.1371/journal.pone.0042808 (PMC3416774; doi:10.1371/journal.pone.0042808)
Supplement: Figure S1 — Immunohistochemistry to assess the HuR and TTP expression in the rabbit abdominal aorta. (DOC) [file pone.0042808.s001.doc]

**Supporting information**

**figure S1:**


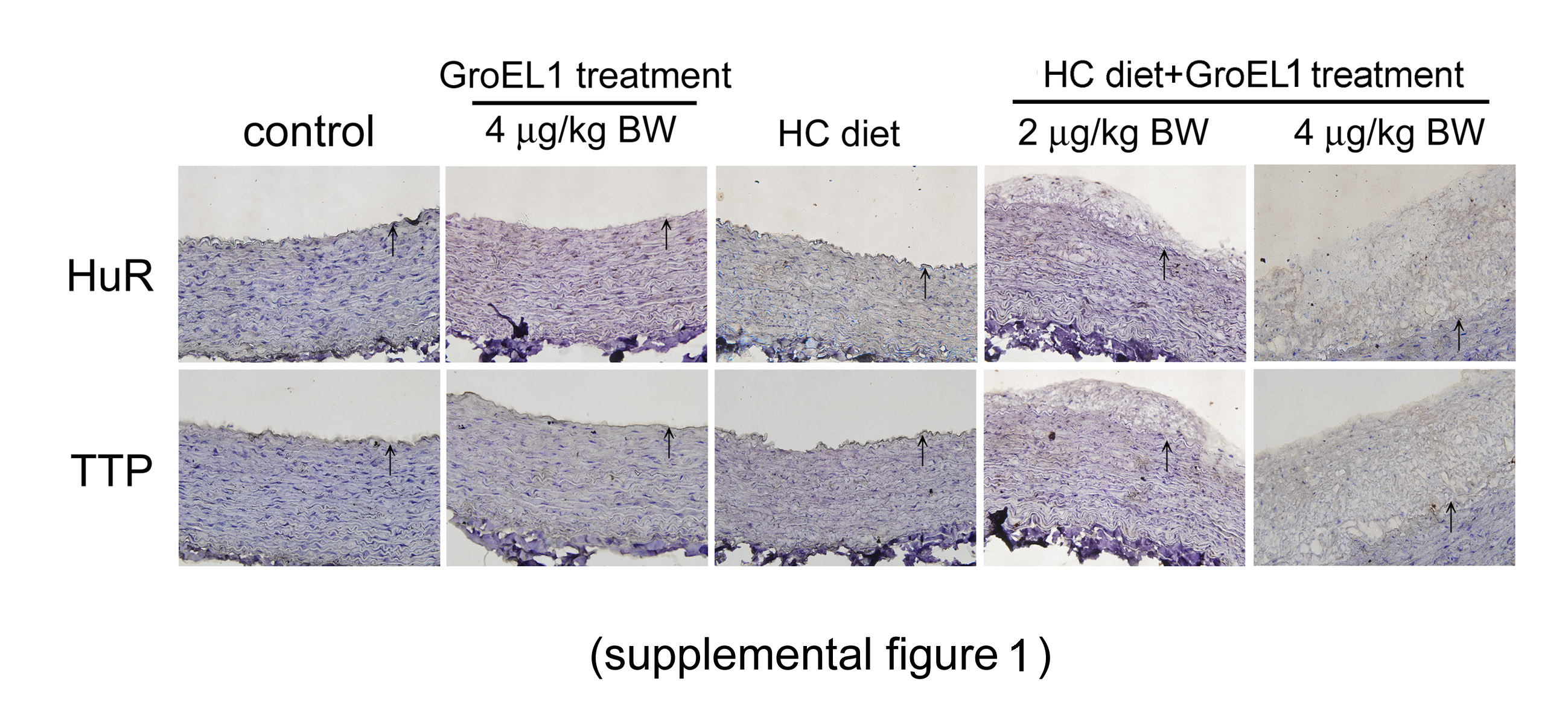


Immunohistochemistry to assess the HuR and TTP expression in the rabbit abdominal aorta. Compared to the control group, the expression of the HuR and TTP remained unchanged in the rabbit aorta following GroEL1 treatment and HC diet. Corresponding hematoxylin staining was used for nucleus identification. The graphs show 100x magnification of slides. The lumen is uppermost in all sections, and the internal elastic laminae is indicated by arrows.
