# Supplementary material for: GroEL1, from Chlamydia pneumoniae, Induces Vascular Adhesion Molecule 1 Expression by p37AUF1 in Endothelial Cells and Hypercholesterolemic Rabbit
Source: PLoS One. 2012 Aug 10;7(8):e42808. doi: 10.1371/journal.pone.0042808 (PMC3416774; doi:10.1371/journal.pone.0042808)
Supplement: Figure S2 — Fluorescent microscopy and western blot analysis demonstrated that the expression level and distribution of HUR and TTP remained unchanged following GroEL1 treatment. (DOC) [file pone.0042808.s002.doc]

**Supporting information**

**figure S2:**


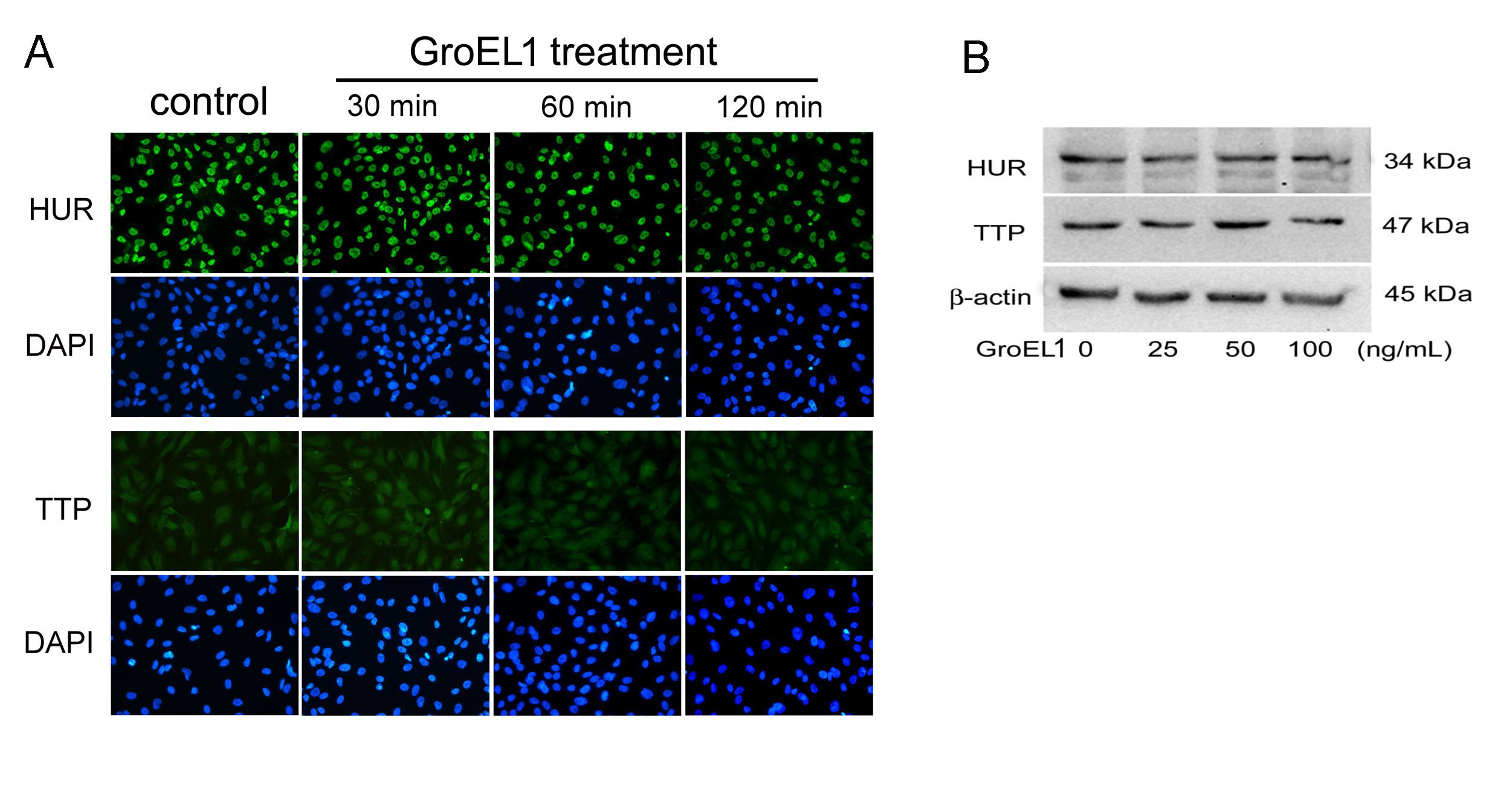


Fluorescent microscopy and western blot analysis demonstrated that the expression level and distribution of HUR and TTP remained unchanged following GroEL1 treatment. (A) HCAECs were treated with 100 ng/mL of GroEL1 for 30-120 minutes. The intracellular HuR and TTP were identified using an immunocytofluorescet assay and observed using fluorescent microscope. DAPI was used to stain the nuclei of the HCAECs cells. (B) HCAECs were treated with 25-100 ng/mL of GroEL1 for 60 minutes, and the total protein was extracted from cells. Western blot analysis was performed for the HuR and TTP expression. β-actin was used as a loading and internal control.
