# Supplementary material for: GroEL1, from Chlamydia pneumoniae, Induces Vascular Adhesion Molecule 1 Expression by p37AUF1 in Endothelial Cells and Hypercholesterolemic Rabbit
Source: PLoS One. 2012 Aug 10;7(8):e42808. doi: 10.1371/journal.pone.0042808 (PMC3416774; doi:10.1371/journal.pone.0042808)
Supplement: Figure S3 — The sequence of the 3′ and 5′_UTR of the VCAM-1 mRNA. (DOC) [file pone.0042808.s003.doc]

**Supporting information**

**figure S3:**


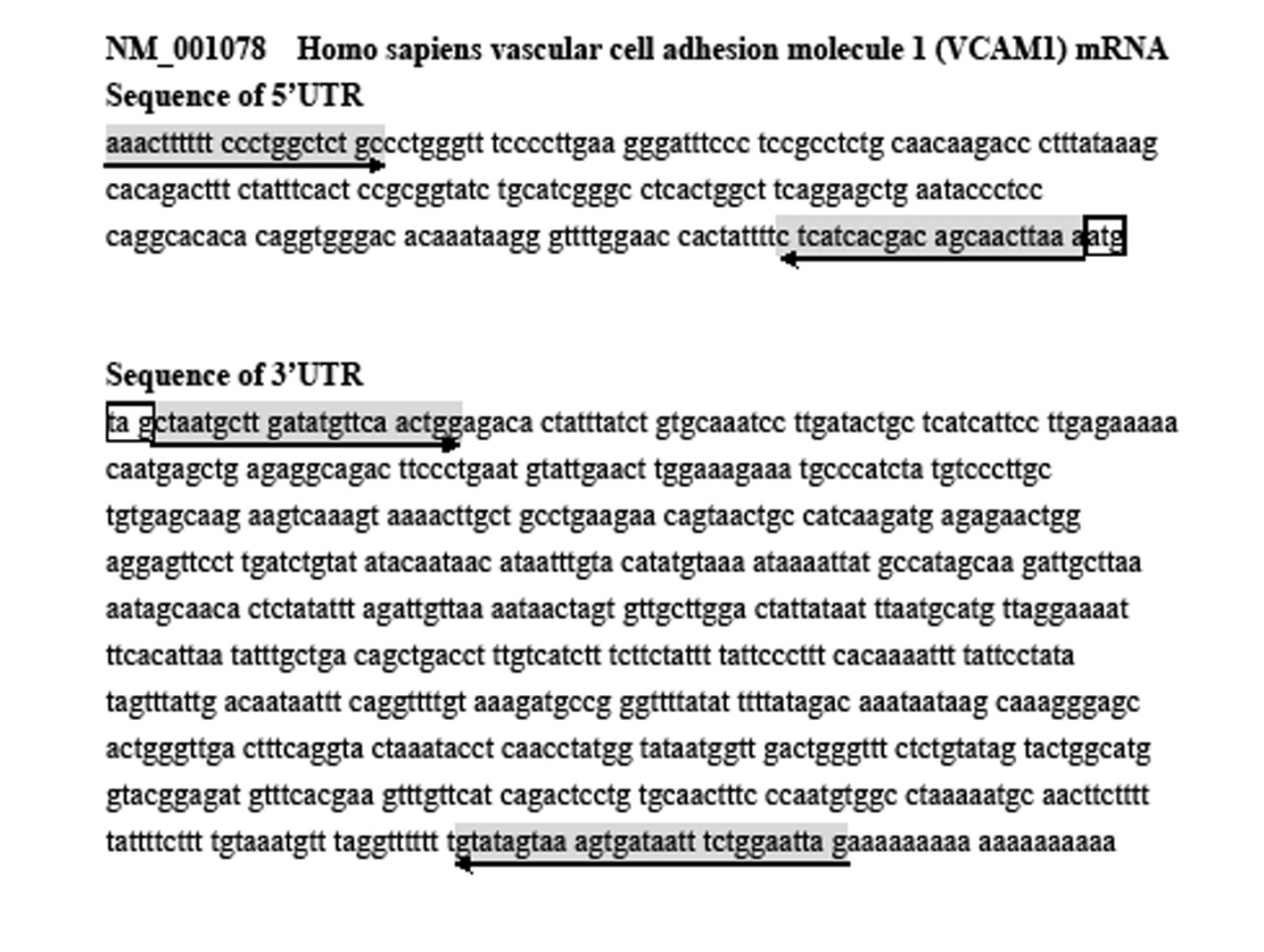


The sequence of the 3’ and 5’ UTR of the VCAM-1 mRNA. The oligonucleotides for the PCR synthesis of the UTR of the VCAM-1 mRNA are marked by the underlining arrows and the gray background.
