# Supplementary material for: GroEL1, from Chlamydia pneumoniae, Induces Vascular Adhesion Molecule 1 Expression by p37AUF1 in Endothelial Cells and Hypercholesterolemic Rabbit
Source: PLoS One. 2012 Aug 10;7(8):e42808. doi: 10.1371/journal.pone.0042808 (PMC3416774; doi:10.1371/journal.pone.0042808)
Supplement: Figure S4 — GroEL1 induces the binding of BAECs/THP-1 cells and impairs the tube formation capacity of the BAECs. (DOC) [file pone.0042808.s004.doc]

**Supporting information**

**figure S4:**

**
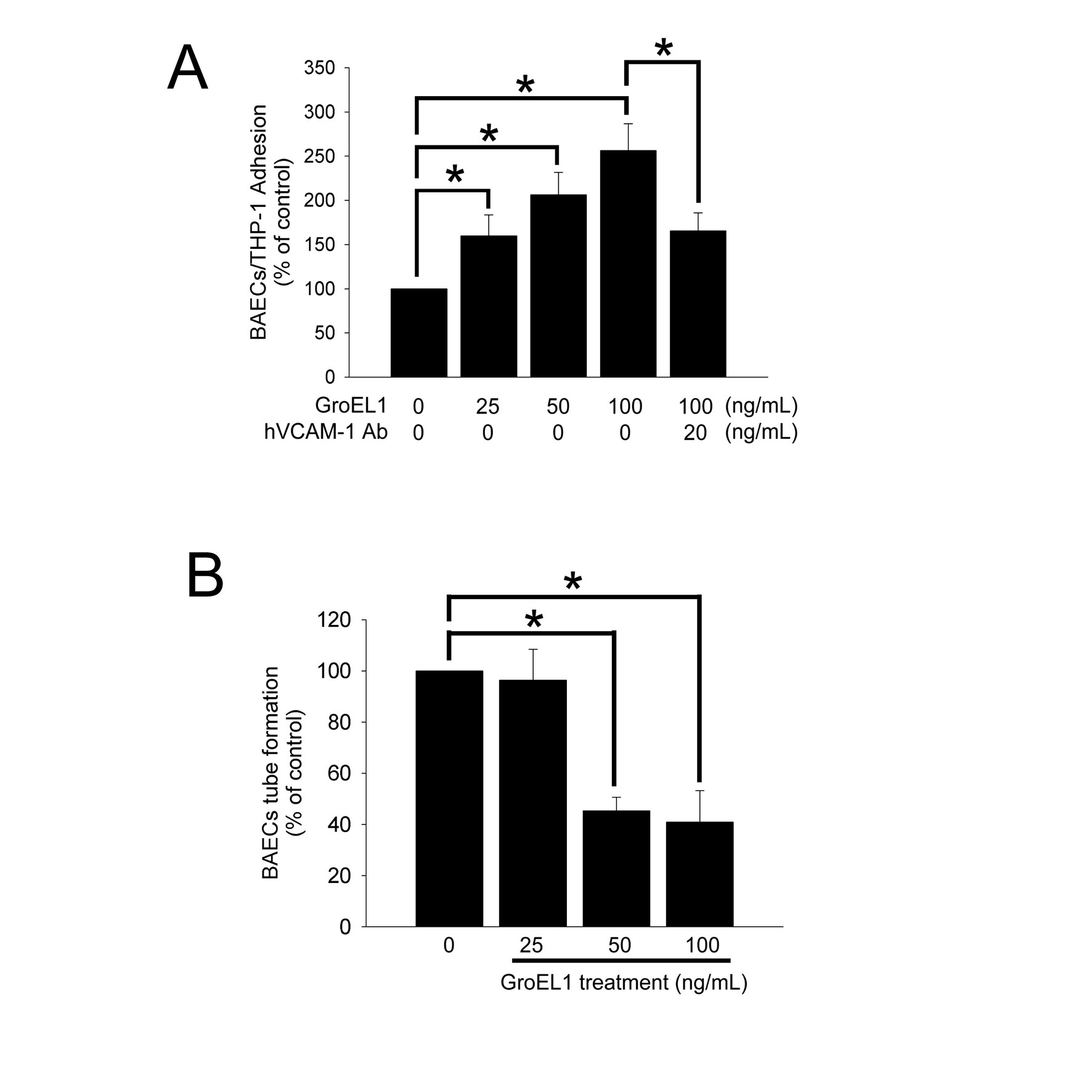
**

GroEL1 induces the binding of BAECs/THP-1 cells and impairs the tube formation capacity of the BAECs. (A) BAECs were pretreated with 25-100 ng/mL of GroEL1 for 24 h or with anti-hVCAM-1 antibodies for 30 min, followed by GroEL1 treatment for 24 h and then co-cultured with THP-1 cells for 1 h. The degree of THP-1 adhesion to the BAECs was counted using a Multilabel Counter Victor^2^. (C) BAECs were pretreated with 25-100 ng/mL of GroEL1 for 48 h. An *in vitro* tube formation assay was performed using ECMatrix gel to investigate the effect of GroEL1 on the BAECs’ lining function. The bar graph demonstrates the tube formation capacity of the GroEL1-treated BAECs. All data represent the results of three independent experiments (mean ± SD; **P* < 0.05 was considered significant and n=3).
