# Supplementary material for: GroEL1, from Chlamydia pneumoniae, Induces Vascular Adhesion Molecule 1 Expression by p37AUF1 in Endothelial Cells and Hypercholesterolemic Rabbit
Source: PLoS One. 2012 Aug 10;7(8):e42808. doi: 10.1371/journal.pone.0042808 (PMC3416774; doi:10.1371/journal.pone.0042808)
Supplement: Figure S5 — GroEL1 increases the production of VCAM-1 mRNA and prolongs the stability of the VCAM-1 mRNA in the BAECs. (DOC) [file pone.0042808.s005.doc]

**Supporting information**

**figure S5:**


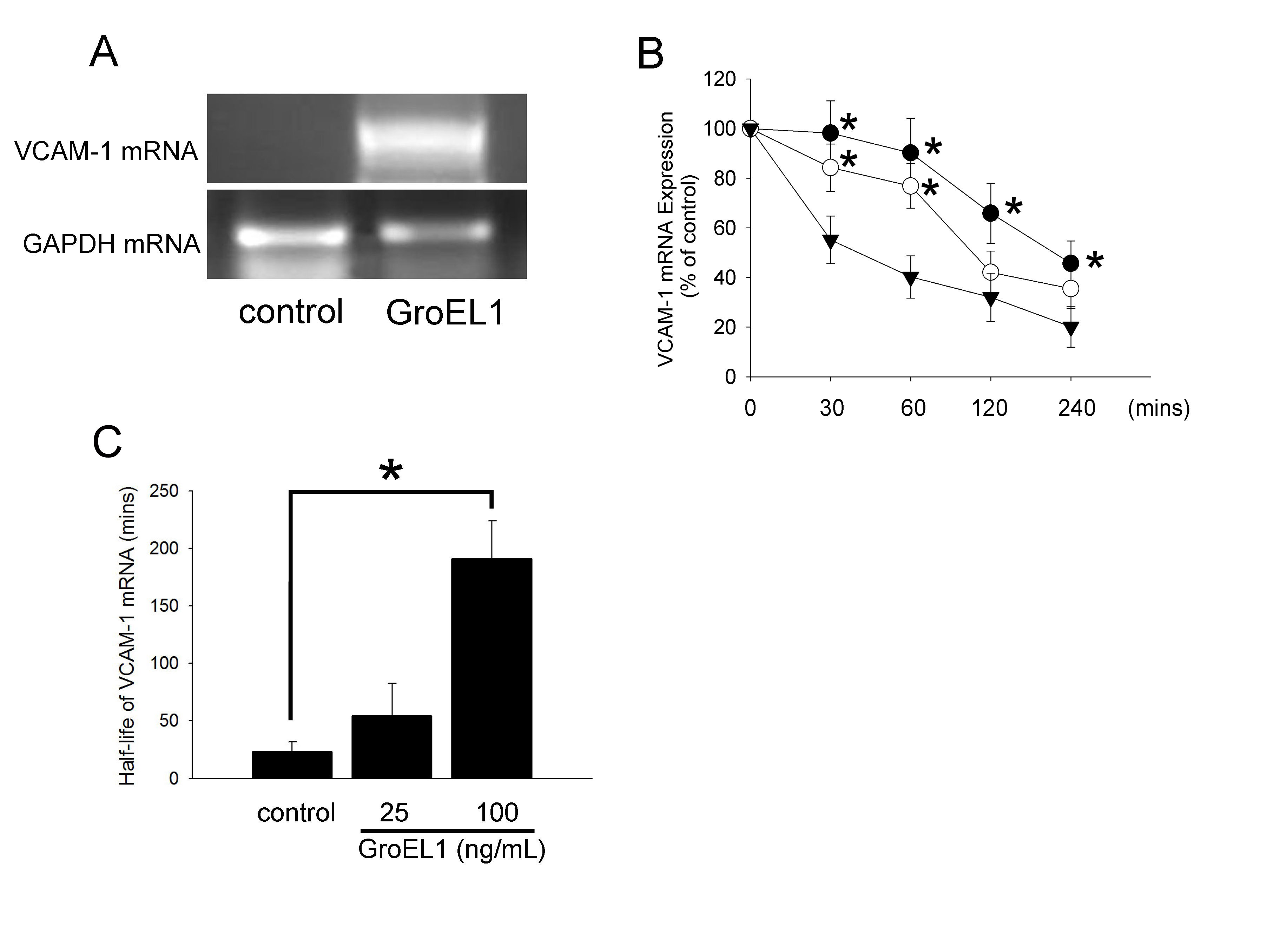


GroEL1 increases the production of VCAM-1 mRNA and prolongs the stability of the VCAM-1 mRNA in the BAECs. (A) Cells were treated for 4 h with 100 ng/mL of GroEL1. Expression of VCAM-1 mRNA was detected by traditional RT-PCR. The total GAPDH mRNA was used as a loading control. (B) Actinomycin D chase experiment was performed to evaluate the stability of the VCAM-1 mRNA. Cells were treated with 25 ng/mL (○) or 100 ng/mL (●) of GroEL1 before actinomycin D treatment for 40 minutes. (▼) The stability of VCAM-1 mRNA was demonstrated in naïve cells. Total RNA was extracted at various time points and quantitative real-time PCR was performed. (C) The half-life of VCAM-1 mRNA were calculated and showed as bar graph. All data represent the results of three independent experiments (mean ± SD; **P* < 0.05 was considered significant compared to control and n=3).
