# Supplementary material for: GroEL1, from Chlamydia pneumoniae, Induces Vascular Adhesion Molecule 1 Expression by p37AUF1 in Endothelial Cells and Hypercholesterolemic Rabbit
Source: PLoS One. 2012 Aug 10;7(8):e42808. doi: 10.1371/journal.pone.0042808 (PMC3416774; doi:10.1371/journal.pone.0042808)
Supplement: Figure S6 — The expression of the 4His-A-AUF1-p37 plasmid, 4His-A-AUF1-p40 plasmid, 4His-A-AUF1-p42 plasmid, and 4His-A-AUF1-p45 plasmid in BAECs. (DOC) [file pone.0042808.s006.doc]

**Supporting information**

**figure S6:**


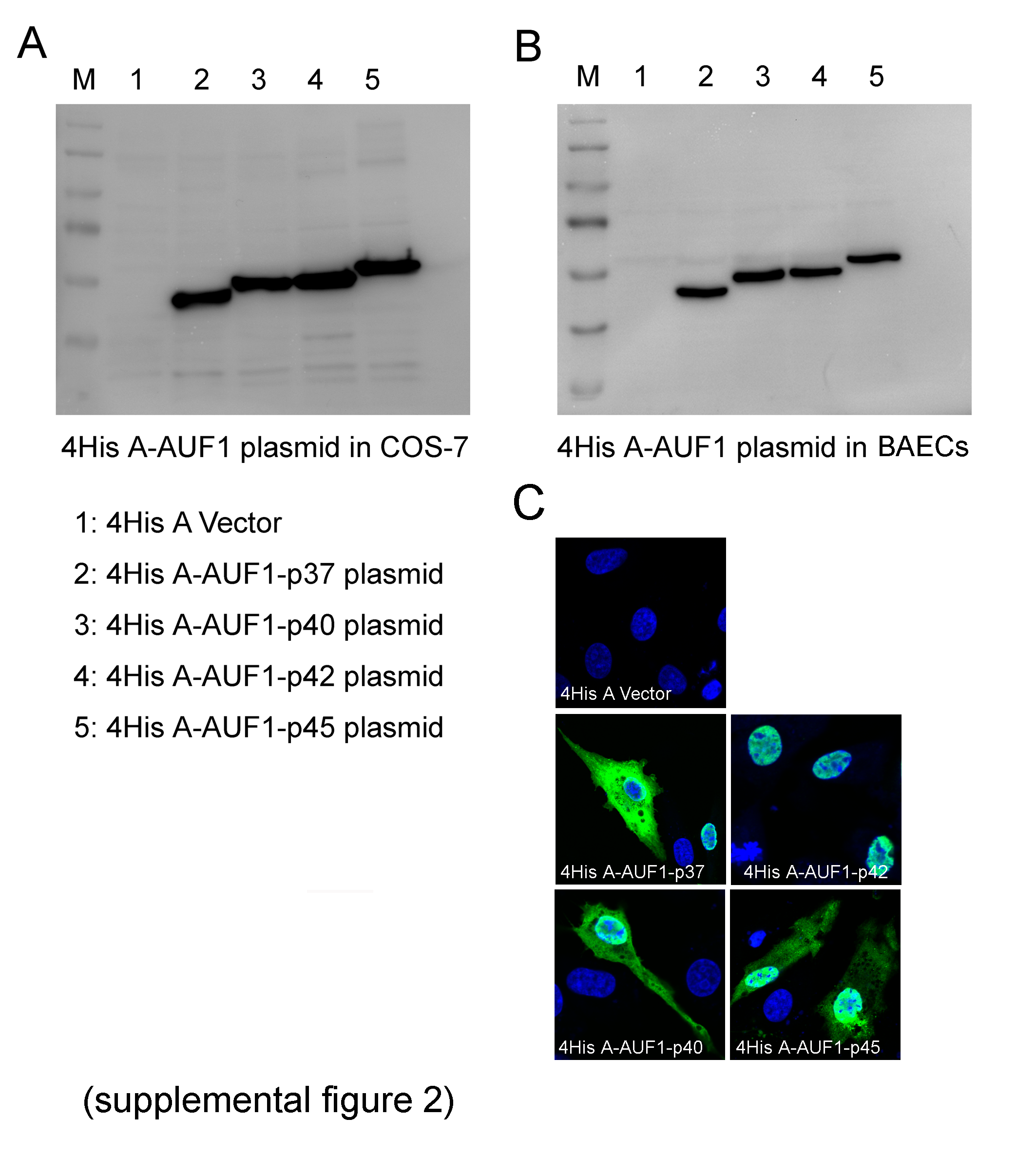


The expression of the 4His-A-AUF1-p37 plasmid, 4His-A-AUF1-p40 plasmid, 4His-A-AUF1-p42 plasmid, and 4His-A-AUF1-p45 plasmid in BAECs. (A) The COS-7 cells were transfected with the 4His-A-AUF1-p37 plasmid, the 4His-A-AUF1-p40 plasmid, the 4His-A-AUF1-p42 plasmid, the 4His-A-AUF1-p45 plasmid, or the 4His-A-empty plasmid. The level of AUF1 isotypes were analyzed using western blotting after transfection for 24 hours. (B and C) The BAECs were transfected with plasmids, and the AUF1 protein expression was identified with western blotting or observed by fluorescent microscope. DAPI was used to stain the nuclei of BAECs.
